# P-1474. Antimicrobial Therapy of *Enterococcus faecium* Bacteremia: A Retrospective Comparison of Outcomes with IV or Oral Antimicrobial Therapy

**DOI:** 10.1093/ofid/ofae631.1644

**Published:** 2025-01-29

**Authors:** Caitlin A Contag, Müge Kalaycıoğlu, Sa Shen, Jorge Salinas, David R Ha, Marisa Holubar, Hector F Bonilla

**Affiliations:** Stanford University, Palo Alto, California; Stanford University, Palo Alto, California; Quantitative Sciences Unit, Stanford, California; Stanford University, Palo Alto, California; Stanford Health Care, Stanford, CA; Stanford University School of Medicine, Stanford, CA; Stanford University School of Medicine, Stanford, CA

## Abstract

**Background:**

Enterococci are one of the most common causes of hospital-associated infections. *Enterococcus faecium* has particularly been associated with prolonged hospitalizations, frequent comorbid conditions, and high rates of mortality.

**Methods:**

This is a single-center, retrospective study of antibiotic use and outcomes in adult patients with *Enterococcus faecium* bacteremia from 2018-2022.

**Results:**

111 patients had monomicrobial *E. faecium* bacteremia. The median age was 58 years (IQR 46.5, 68.5 years) and 63/111 (57%) of patients were male. Of isolates that underwent susceptibility testing, 15/109 (14%) were susceptible to ampicillin and 33/109 (30%) were susceptible to vancomycin. The median length of hospitalization for *E. faecium* bacteremia was significantly longer than for *E. faecalis* bacteremia (26 days versus 12 days, p< 0.01). Nearly a third of patients with *E. faecium* bacteremia died or were discharged to hospice during their index hospitalization (34/111, 31%) compared with 24/136 (18%) of patients with *E. faecalis* bacteremia (p=0.017). The 30-, 60-, and 90-day mortality was 25%, 32%, and 36% for *E. faecium* bacteremia and 15%, 18%, and 25% for *E. faecalis* bacteremia.

Most patients with *E. faecium* bacteremia were treated with only intravenous (IV) antibiotics. Of the patients who received *E. faecium* directed antimicrobial therapy, 35/106 (33%) received a combination of IV and oral antimicrobials. Patients treated with IV therapy alone had a higher rate of mortality at 30, 60, and 90 days than those treated with a combination of oral and IV therapy (IV versus oral: 34% versus 3% 30-day mortality, 39% versus 15% 60-day mortality, and 44% versus 18% 90-day mortality). *E. faecium* bacteremia recurrence within 5 to 90 days occurred in 15/111 (14%) patients. Of these patients, 10/15 received only IV antimicrobials, whereas 5/15 received some oral therapy.

**Conclusion:**

*E. faecium* bacteremia was associated with prolonged hospitalizations and high rates of mortality when compared with

*E. faecalis* bacteremia. Patients treated with a combination of oral and IV therapy did not have an increased risk of mortality or bacteremia recurrence. The difference in outcomes is likely related to severity of disease and comorbid factors affecting clinicians’ antimicrobial choice.

**Disclosures:**

**All Authors**: No reported disclosures

